# Is the Use of Dialysis Associated With an Increased Risk of Death in COVID-19-Related Acute Kidney Injury?

**DOI:** 10.7759/cureus.32373

**Published:** 2022-12-10

**Authors:** Ghita El Bardai, Salma Sqalli Houssaini, Basmat Amal Chouhani, Nadia Kabbali, Tarik Sqalli Houssaini

**Affiliations:** 1 Nephrology, Dialysis, and Transplantation Department, Hassan II University Hospital, Fez, MAR; 2 Laboratory of Epidemiology and Health Science Research (ERESS), Faculty of Medicine-Fez, Sidi Mohammed Ben Abdelalh University, Fez, MAR

**Keywords:** mortality, hd ( hemodialysis ), ards (acute respiratory distress syndrome), acute kidney injury, covid-19

## Abstract

Introduction: Acute kidney injury (AKI) is frequently reported in the setting of severe acute respiratory syndrome coronavirus 2 (SARS‑CoV‑2) infection. The aim of our work is to evaluate the impact of acute dialysis use on mortality in patients with AKI during the coronavirus disease 2019 (COVID-19) pandemic.

Methods: This is a retrospective study conducted in the Hassan II University Hospital of Fez, Morocco. From July 2020 to December 2021, we included all patients admitted to a COVID-19 unit with acute kidney injury defined according to Kidney Disease Improvement Global Outcomes 2012 (KDIGO 2012) criteria. Our patients were older than 18 years, and SARS-CoV-2 infection was confirmed by a positive RT-PCR test or thoracic CT scan imaging. Patients with end-stage renal disease (ESRD) and pregnant women were excluded from our study.

Results: The total number of patients hospitalized in the COVID-19 unit during the study period was 2560, including 206 in an intensive care setting. We included 61 patients with AKI, with an incidence in the intensive care unit (ICU) setting of 15.5%. Eighty percent of patients had respiratory distress on admission, which was the main reason for consultation. Stage 1 AKI was found in 1.6% of patients, 25.8% had stage II AKI, and 72.6% had KDIGO stage 3 AKI. The main etiology of AKI was acute tubular necrosis. Lung involvement secondary to infection was severe in 18 patients; 21 had moderate involvement. In our study, twenty-one of our patients (34.4%) were hospitalized in an ICU. Thirteen of our patients were intubated (21.1%). Twenty-one (34.4%) patients were hemodynamically unstable and were put on vasoactive drugs. Twenty-three (37.7%) of our patients received at least one session of conventional acute hemodialysis with an average duration of 2.1 hours ± 0.9 (1-3.5). The indication was overload (27%), severe metabolic acidosis (1.6%), threatening hyperkalemia (1.6%), and symptomatic hyperuremia (62%). The evolution was marked by a return to baseline renal function in two patients, partial improvement in 35 of them at discharge, and no improvement in 24 patients. We recorded a death rate of 34.4% (n=21). In a univariate analysis, we compared the demographic, clinical, paraclinical, and dialytic characteristics of the dialysis and non-dialysis groups. There was a significant difference between unstable, intubated patients and those hospitalized in the ICU in the dialysis group, with respective p-values of p=0.0001, p=0.0001, and p=0.01. We noticed there were more deaths in the dialysis group than in the non-dialysis group; this difference was statistically significant with a p-value of 0.005. In multivariate analysis, a logistic regression model was performed to test the relationship between dialysis and COVID-19 mortality while adjusting for other co-factors. The final model did not show a significant association between dialysis and mortality (p = 0.150, OR: 2.578 [0.710-9.364]). The only factor that remained independently significant was admission to the intensive care unit (p = 0.004, OR: 6.732 [1.847-24.540]).

Conclusion: AKI is a frequently encountered complication in patients with COVID-19, especially those hospitalized in the ICU. In the context of the SARS-CoV-2 infection, the use of at least one dialysis session seems to represent an excess risk of mortality related to AKI.

## Introduction

In late 2019, Wuhan Province in China was the site of the emergence of a new disease responsible for life-threatening viral lung disease. This severe acute respiratory syndrome coronavirus 2 (SARS-CoV-2) infection has been referred to as COVID-19 disease; it represents the seventh form of the coronavirus family infecting the human species and causing a global pandemic [1]. Since its emergence, nearly 571 million reported cases and 6.3 million deaths have occurred worldwide until July 2022. In Morocco, 1.1 million new cases have been registered, with 16,000 deaths throughout the kingdom [2].

Although acute respiratory failure is the most severe and widespread organ dysfunction, acute kidney injury (AKI) is frequently reported in the setting of a SARS-CoV-2 infection. Indeed, several cases of COVID-19-related acute kidney injury have been reported in the literature, with 5% to 15% of total cases requiring continuous renal replacement therapy (RRT) and 5% to 58% of critically ill patients requiring continuous RRT [3,4]. Many hypotheses have been put forward to explain this kidney damage, including hypoperfusion renal, sepsis, and thunderstorm cytokine or direct virus toxicity on renal tubular cells [5]. An association between AKI and in-hospital mortality was found, with the risk of death increasing with the severity of the AKI [6].

The aim of the present work is to evaluate the impact of the use of acute dialysis on the mortality of patients with AKI during a COVID-19 pandemic at the Hassan II University Hospital (CHU) of Fez, Morocco.

## Materials and methods

Study design

This is a single-center retrospective study carried out at the Hassan II University Hospital, Fez, Morocco. The study was conducted from July 2020 to December 2021.

Patients

We included patients admitted to the COVID-19 unit with acute kidney injuries. We included all adult patients (age ≥18 years old) with confirmed laboratory SARS-CoV-2 infection via reverse transcription polymerase chain reaction (RT-PCR) testing of nasopharyngeal swabs. Patients with end-stage renal disease (ESRD) and pregnant women were excluded from our list.

Definitions

CT-diagnosed SARS-CoV-2-related lung involvement was classified according to a five-stage visual classification based on the percentage of lung involvement: absent or minimal (<10%), moderate (10-25%), extensive (25-50%), severe (50-75%), or critical (>75%) [7]. AKI was our primary endpoint and was defined according to the Kidney Disease Improvement Global Outcomes 2012 (KDIGO 2012) criteria: an increase in creatinine of ≥0.3 mg/dL within 48 hours or ≥50% within seven days, or a urine output of <0.5 mL/kg/hour for >6 hours [8]. For patients with a previous serum creatinine within 7 to 365 days before admission, the most recent serum creatinine value was considered the baseline creatinine. For patients without baseline creatinine in the 7 to 365 days prior to admission, a GFR calculation by the MDRD formula was performed; in addition, AKI was categorized according to the three severity stages based on KDIGO recommendations [5]. The indication for acute RRT was established in the presence of several situations: hyperkalemia refractory to drug measures, severe metabolic acidosis, severe volume overload refractory to diuretic treatment, and symptomatic uremic syndrome [9]. Intensive care unit patients were defined as patients in need of intubation or non-invasive ventilation (NIV). Non-stable patients were defined as patients who were hemodynamically unstable and put on vasoactive drugs.

Data collection

All patient data were obtained from the digital platform of the Hassan II University Hospital and then recorded on previously established data sheets with the following headings: demographic characteristics, comorbidities, clinical signs, paraclinical data (biological and CT scan), treatment modalities (hemodialysis, oxygen by nasal cannula, high flow cannula, non-invasive positive pressure ventilation or mechanical ventilator support, the use of vasopressors support), renal evolution at discharge (recovery of renal function, return to baseline creatinine levels, no improvement), and death.

Statistical analysis

Data were collected using Excel software (Microsoft Excel, Microsoft® Corp., Redmond, WA) and analyzed using SPSS v20 software (IBM Corp., Armonk, NY). A descriptive analysis was performed, quantitative variables were expressed as mean ± standard deviation (SD), and qualitative variables were expressed as percentages. In order to determine the mortality risk factors related to COVID-19, the comparison of two means was carried out using the Student test, and the comparison of two percentages was carried out using the Chi² test. In multivariate analyses, a logistic regression model was performed to test the relationship between dialysis and COVID-19 mortality while adjusting for other co-factors. The level of significance adopted was p < 0.05.

## Results

The total number of patients hospitalized in the COVID-19 unit during the study period was 2560, including 206 in an intensive care setting. Our study included 61 patients with AKI, the majority of whom were men (sex ratio F/M 0.4) with a mean age of 61.2 years (±17.4 years). Hypertension was noted in 62% of the patients, 33% of the patients were type 2 diabetics, 4.9% had heart disease, 23% were followed up for CKD (not yet terminal), and one of our patients was a carrier of chronic pneumonia.

In our series, 83.3% of the patients had a positive SARS-CoV-2 RT-PCR on admission. Respiratory distress was the main reason for consultation in 80% of patients, and 56.6% were found to have a fever. Three of our patients had signs of overload, while diuresis was preserved in most of them, i.e., 75.4%. Oligo-anuria was seen in 24.6% of patients.

The biological assessment showed that 1.6% of patients had stage I AKI, 25.8% had stage II AKI, and 72.6% had stage III AKI. Hyperkalemia was observed in 16.3% of our patients with a mean of 6.3 mEq/L ± 0.6 (5.6-7.8 mEq/L), hypernatremia in 11.4% of our patients with a mean of 154.3 mmol/L ± 1.1 (152-161 mmol/L), and hyponatremia in 17 patients varying between 119 and 134 mmol/L with a mean of 125.5 ± 4.5 mmol/L. Leukocytosis with predominantly neutrophils was observed in 56.6%, 36.6% had lymphopenia, and CRP varied between 55 and 433 mg/l with a mean of 180.2 ± 117. On imaging, lung involvement secondary to infection was severe in 18 patients, 21 had moderate involvement, and 22 had minimal involvement on non-contrast CT chests, according to a five-stage visual classification based on the percentage of lung damage [4].

Twenty-one of our patients (34.4%) were hospitalized in an intensive care unit (ICU). Management included the use of isotonic saline and/or Ringer's lactate for patients with clinical (the presence of signs of dehydration) and echographic (cardiac ultrasound: collapsed inferior vena cava, low filling pressure) signs of hypovolemia. Those with signs of volume overload received loop diuretics during their stay. Thirteen of our patients were intubated (21.1%), ten were under non-invasive ventilation (16.7%), and 37 were put on oxygen therapy (61.7%). Twenty-one (34.4%) patients were hemodynamically instable and were put on vasoactive drugs. Twenty-three of our patients (37.7%) received at least one session of conventional acute hemodialysis with an average duration of 2.1 hours ± 0.9 (1-3.5). The indications were overload (27%), severe metabolic acidosis (1.6%), threatening hyperkalemia (1.6%), and symptomatic hyperuremia (62%). The evolution was marked by a return to baseline renal function in 2 patients, partial improvement in 35 of them at discharge, and no improvement in 24 patients. We recorded a death rate of 34.4% (n=21).

In a univariate analysis, we compared the demographic, clinical, paraclinical, and dialytic characteristics of the dialysis and non-dialysis groups. No significant difference was found concerning the stage of renal failure or the severity of pulmonary damage. However, there was a significant difference between unstable, intubated patients and those hospitalized in the ICU in the dialysis group, with a respective p of (p = 0.0001, p=0.0001, p=0.01). We noticed there were more deaths in the dialysis group than in the non-dialysis group; this difference was statistically significant with a p = 0.005 (Table 1).

**Table 1:** Comparison of demographic, clinical, and paraclinical characteristics of dialysis and non-dialysis patients. NS: non significant; p > 0.05; RRT: renal replacement therapy; AKI: acute kidney injury.

Population characteristics	RRT n = 23	No RRT n= 38	P-value
Mean age ±SD	62.3 ± 17.4	59.6 ± 12.9	NS
Sex ratio W/M	0.3 (9/23)	0.2 (9/38)	NS
High blood pressure n (%)	18 (78)	20 (52)	NS
Type 2 diabetes n (%)	11 (47)	9 (23)	NS
Chronic kidney disease n (%)	8 (34)	6 (15)	NS
Conserved diuresis n (%)	13 (16.4)	33 (86)	NS
Oligo-anuria n (%)	10 (43)	5 (13)	NS
AKI stage I n (%)	None	1 (2.6)	NS
AKI stage II n (%)	1 (1.6)	14 (37)	NS
AKI stage III n (%)	22 (95)	23 (60)	NS
Potassium levels (mean ±SD range)	4.1 ±1.6 (2.4-7.8)	3.8 ± 0.5 (3.1-5.9)	NS
Alkali reserve levels (mean ± SD range)	11 ± 5.1 (5-18)	17 ± 5.7 (8-24)	NS
Severe pulmonary lesions on CT scan n (%)	10 (43)	8 (21)	NS
Intensive care unit n (%)	14 (60.9)	7 (18.4)	0.01
Death n (%)	13 (56.5%)	8 (21.1)	0.005
Hemodynamic instability	15 (65.2)	6 (15.8)	0.0001
Mechanical ventilation support	11 (47.8)	2 (5.4)	0.0001

In multivariate analysis, a logistic regression model was performed to test the relationship between dialysis and COVID-19 mortality while adjusting for other co-factors. The final model did not show a significant association between dialysis and mortality (p = 0.150, OR = 2.578 [0.710-9.364]). The only factor that remained independently significant was admission to the intensive care unit (p = 0.004, OR = 6.732 [1.847-24.540]).

## Discussion

In our study, of all patients with AKI associated with COVID-19 damage, more than one-third required acute dialysis, which was associated with high mortality in these patients (34.4%). The majority of survivors were not able to regain their baseline renal function upon discharge from the hospital.

Data from the literature suggest a causal link between acute respiratory distress syndrome (ARDS) of any etiology and AKI. In a large cohort of more than 1800 intensive care patients with ARDS, nearly half had AKI compared to 27.4% of non-ARDS intensive care patients [10]. Although the current COVID-19 pandemic is caused by a coronavirus similar to the 2005 ARDS-1 coronavirus outbreak, the reported incidence of AKI associated with COVID-19 disease appears to be more frequent; AKI occurred in 1835 patients (46%), and 347 (19%) of those with AKI required dialysis [11,12]. This incidence would be higher than that reported in patients hospitalized with community-acquired pneumonia (34%) in the United States [12].

In the Chinese cohorts of SARS-CoV-2 infection, the prevalence of AKI appeared to be negligible or non-existent [13]. The progression of the pandemic worldwide has subsequently been described in a large literature, which has shed new light on the incidence of AKI, especially in patients hospitalized in ICUs. It is estimated to be around 6%, although there is great variability between studies [14-15]. The characteristics and severity of the patients in these different cohorts, in addition to variations in patient management, may have an important influence on the development of AKI, thus explaining these differences in prevalence [3]. In addition, differences in gene expression at the ethnic level, with an angiotensin-converting enzyme 2 (ACE2) receptor expression polymorphism between Caucasian and Asian patients, suggest that Western populations may be at greater risk of developing AKI [16].

Chronic kidney disease was independently associated with severe AKI in a study performed at the Mount Sinai Health System in New York. As reported in the literature, patients admitted to the ICU with a higher incidence of AKI are those with multiple comorbidities [12].

In our study, we report a high mortality rate in our patients. Indeed, there is an association between AKI and in-hospital mortality in subjects with SARS-CoV-2 infection, with a risk of death that increases with the severity of AKI, after adjustment for age, severity, and comorbidities of the patients [6].

Given the high incidence of AKI and the lack of recovery of renal function in a proportion of cases [12], knowledge of the pathophysiological mechanisms of COVID-19-related AKI is essential to ensure better management and reduce the potential risk of chronicity. Several hypotheses have been put forward to explain this renal damage, including renal hypoperfusion related to mechanical ventilation in ICU patients, sepsis and cytokine storms, or direct toxicity of the virus on tubular cells [5]. Pathophysiologically, co-expression of ACE 2 receptors and transmembrane protease serine 2 (TMPRSS2) is required for virus entry into the host cell. Direct renal involvement of the virus is strongly suspected due to the high concentration of these receptors in the kidney, especially in the proximal tubule and podocytes [3]. An immunohistochemical study of an autopsy report of 26 patients with SARS-CoV-2 confirmed the presence of the virus in the proximal convoluted tubule with loss of the brush border and even cell necrosis [17]. Proximal tubulopathies are characterized by polyuria and increased excretion of sodium, potassium, chlorine, bicarbonates, glucose, and low molecular weight proteins, which can lead to dehydration, hyponatremia, hypokalemia, hypophosphatemia, and hyperchloremic acidosis, as well as renal failure. More recently, a few cases of glomerulopathy related to podocyte damage have been described, although it is not yet known whether they are related to direct virus toxicity or to the cytokine storm [6].

More than half of our patients with COVID-19-associated AKI did not return to their baseline creatinine value. The low recovery rate is expected given the overall severity of AKI, which is also explained by the presence of extensive acute tubular injury on pathological examination as well as potential microthrombi in many patients with COVID-19 [17,18]. The use of acute dialysis is one of the predictors of the lack of recovery of baseline renal function in these patients. The incidence of the use of dialysis in the ICU for COVID-19 patients is significant; it ranges between 17 and 21% [15], and it is accompanied by a high risk of death. Our study showed a trend toward an increased risk of mortality in dialysis patients with an odds ratio of 2.578 (0.710-9.364) without being statistically significant. This may be explained by a lack of statistical power given our small sample size. Our results were confirmed by a large American cohort of 4221 patients in a study published in the American Journal of Kidney Diseases, which showed high mortality in patients with COVID-19 who had an AKI and were dialyzed, i.e., 67% [19]. In another study including 3993 patients hospitalized for COVID-19 in New York, AKI was revealed in 1835 (46%) patients, among whom 19% required dialysis, and half of those who were dialyzed died in the hospital [12]. It should be noted that the English guidelines recommend following the usual RRT strategy in the ICU [20].

## Conclusions

In conclusion, AKI is a complication frequently encountered in patients with COVID-19, especially those hospitalized in intensive care units. In this study conducted at the Hassan II University Hospital in Fez, we described a very high incidence of AKI, and the use of at least one dialysis session seems to represent an excess risk of mortality related to AKI. We found that more than half of the patients did not recover to have baseline renal function at discharge, hence needing long-term follow-up and further investigation.
